# Mitochondrial DNA in bronchoalveolar lavage fluid is associated with the prognosis of idiopathic pulmonary fibrosis: a single cohort study

**DOI:** 10.1186/s12931-024-02828-9

**Published:** 2024-05-10

**Authors:** Jun Fukihara, Koji Sakamoto, Yoshiki Ikeyama, Taiki Furukawa, Ryo Teramachi, Kensuke Kataoka, Yasuhiro Kondoh, Naozumi Hashimoto, Makoto Ishii

**Affiliations:** 1 Department of Respiratory Medicine and Allergy, Tosei General Hospital, Seto, Aichi Japan; 2 Department of Respiratory Medicine, Nagoya University Graduate School of Medicine, 65 Tsurumai-Cho, Showa-Ku, Nagoya, Aichi Japan; 3 Medical IT Center, Nagoya University Hospital, Nagoya, Aichi Japan; 4 Department of Respiratory Medicine, Fujita Health University School of Medicine, Toyoake, Aichi Japan

**Keywords:** Acute exacerbation, Bronchoalveolar lavage, Droplet digital PCR, Idiopathic pulmonary fibrosis, Interstitial lung disease, Nucleolar DNA, Mitochondrial DNA

## Abstract

**Background:**

Extracellular mitochondrial DNA (mtDNA) is released from damaged cells and increases in the serum and bronchoalveolar lavage fluid (BALF) of idiopathic pulmonary fibrosis (IPF) patients. While increased levels of serum mtDNA have been reported to be linked to disease progression and the future development of acute exacerbation (AE) of IPF (AE-IPF), the clinical significance of mtDNA in BALF (BALF-mtDNA) remains unclear. We investigated the relationships between BALF-mtDNA levels and other clinical variables and prognosis in IPF.

**Methods:**

Extracellular mtDNA levels in BALF samples collected from IPF patients were determined using droplet-digital PCR. Levels of extracellular nucleolar DNA in BALF (BALF-nucDNA) were also determined as a marker for simple cell collapse. Patient characteristics and survival information were retrospectively reviewed.

**Results:**

mtDNA levels in serum and BALF did not correlate with each other. In 27 patients with paired BALF samples obtained in a stable state and at the time of AE diagnosis, BALF-mtDNA levels were significantly increased at the time of AE. Elevated BALF-mtDNA levels were associated with inflammation or disordered pulmonary function in a stable state (*n* = 90), while being associated with age and BALF-neutrophils at the time of AE (*n* = 38). BALF-mtDNA ≥ 4234.3 copies/µL in a stable state (median survival time (MST): 42.4 vs. 79.6 months, *p* < 0.001) and ≥ 11,194.3 copies/µL at the time of AE (MST: 2.6 vs. 20.0 months, *p* = 0.03) were associated with shorter survival after BALF collection, even after adjusting for other known prognostic factors. On the other hand, BALF-nucDNA showed different trends in correlation with other clinical variables and did not show any significant association with survival time.

**Conclusions:**

Elevated BALF-mtDNA was associated with a poor prognosis in both IPF and AE-IPF. Of note, at the time of AE, it sharply distinguished survivors from non-survivors. Given the trends shown by analyses for BALF-nucDNA, the elevation of BALF-mtDNA might not simply reflect the impact of cell collapse. Further studies are required to explore the underlying mechanisms and clinical applications of BALF-mtDNA in IPF.

**Supplementary Information:**

The online version contains supplementary material available at 10.1186/s12931-024-02828-9.

## Introduction

Idiopathic pulmonary fibrosis (IPF) is a chronic, progressive, and fibrotic lung disease with a median survival time (MST) of 3–5 years [1]. Acute exacerbation (AE) is a devastating complication of IPF with an MST of only 3 months [2]. Although some profibrotic growth factors, cytokines, and chemokines have been identified as contributing to the disease, its pathogenesis remains not fully understood. Given the poor prognosis of patients with IPF and AE-IPF, it is crucial to gain a thorough understanding of their pathogenesis and identify clinically important biomarkers.

Extracellular mitochondrial DNA (mtDNA) is one of the danger-associated molecular pattern molecules (DAMPs) released from stressed cells, binds to pathogen recognition receptors, activates immune systems, and induces an inflammatory response. In IPF, mitochondrial dysfunction and metabolic changes induced by the profibrotic stimuli and mechanical cues are assumed to trigger the release of mtDNA from lung cells such as fibroblasts [3]. and alveolar epithelial cells [4]. In accordance with this, mtDNA levels are elevated in both serum [3–7] and bronchoalveolar lavage fluid (BALF) [3, 4] in IPF patients. While serum mtDNA levels are associated with survival time [3, 7], disease severity or progression [4, 5, 7], and the occurrence of AE [6], the clinical significance of mtDNA in BALF (BALF-mtDNA) has not been well studied.

In the past 10–20 years, the technique of digital polymerase chain reaction (PCR), a refinement of conventional PCR methods, has become increasingly used for experimental and clinical purposes. Droplet-digital PCR (ddPCR) is one of those novel techniques. This enables precise detection of very small number of targets compared to conventional methods and absolute quantification without need for a standard curve [8, 9] using crude clinical samples without the time- and labour-consuming DNA extraction steps [10].

In this study, we established a protocol for measuring BALF-mtDNA using ddPCR and evaluated its clinical significance in IPF including the value as a possible prognostic biomarker in both stable and AE states.

## Methods

### Study population and data collection

From August 2009 to May 2017, we collected BALF samples from 101 IPF patients who underwent bronchoalveolar lavage (BAL) at Tosei General Hospital. Of those, 90 patients provided BALF samples during a stable state, 38 patients provided at the time of their first AE, and 27 out of them underwent BAL both in a stable state and at the time of AE. A subset of these patients also provided blood samples. During a stable state, 57 out of 90 patients provided blood samples within 3 months before or after undergoing BAL. At the time of the first AE, 36 out of 38 patients provided blood samples at the same time as BAL.

In all patients, the validity of the IPF diagnosis was reconfirmed based on the latest guidelines [11] through multidisciplinary discussions. The AE diagnosis was also reconfirmed as a clinical event meeting the criteria described in an international report published in 2016 [2]. The treatment for AE followed a standardised protocol: methylprednisolone pulse therapy (1 g/day for 3 days/week) for two cycles, followed by 1 mg/kg/day of methylprednisolone. The steroid dosage was gradually tapered to 10 mg/day of oral prednisolone within 4–6 weeks after initiating treatment. Cyclosporine or tacrolimus was administered concurrently. Prednisolone and immunosuppressants were continued for at least a few months post-recovery.

Patient characteristics and test results were collected retrospectively from clinical charts. Blood test and pulmonary function test results were recorded within 3 months before BAL for patients in a stable state. Forced vital capacity (FVC) and diffusing capacity of the lung for carbon monoxide (D_L_CO) were expressed as a percentage of predicted values. To calculate the partial pressure of arterial oxygen (PaO_2_)/fraction of inspiratory oxygen (FiO_2_), FiO_2_ was roughly assumed to be 0.21 + 0.04 × oxygen flow (L/min) when using a nasal cannula, and as 0.60 + 0.10 × (oxygen flow – 6 L/min) when using a non-rebreather mask. For data at the time of AE-IPF, baseline FVC and D_L_CO had been recorded within 6 months prior to the diagnosis of AE. The final follow-up date of this study was 30th April 2022.

### Sample collection

BALF and blood samples were collected from patients who provided informed written consent in accordance with the Declaration of Helsinki. BAL was carried out according to the standardised protocol [12].

See the Supporting Information for the detail protocol for processing of BALF and blood and subsequent ddPCR.

### Statistical analysis

Continuous variables were presented as the median with an interquartile range due to a non-normal distribution, and Wilcoxon signed-rank test and Mann–Whitney’s *U* test were used for inter-group comparisons, as appropriate. Categorical variables were summarized by number and percentage. Spearman’s rank correlation test was applied to evaluate the correlation between two continuous variables. Benjamini and Hochberg’s method [13] was used to adjust the false discovery rate for multiple testing.

We measured survival time from the date of BAL to the date of death. Time-dependent receiver operating characteristic (ROC) curve analyses were performed to determine the optimal cut-off values for BALF-mtDNA and BALF- nucDNA to predict survival time and determine the area under the curve (AUC). Cox proportional hazards models were used to evaluate the associations between variables and survival time, adjusting for known demographic factors being associated with survival time.

We used a two-sided test for all statistical analyses and considered p-values < 0.05 as statistically significant. The analyses were conducted with R commander (The R Foundation for Statistical Computing, Vienna, Austria).

## Results

### Factors correlated with BALF-mtDNA and BALF-nucDNA

**Fig. 1:**
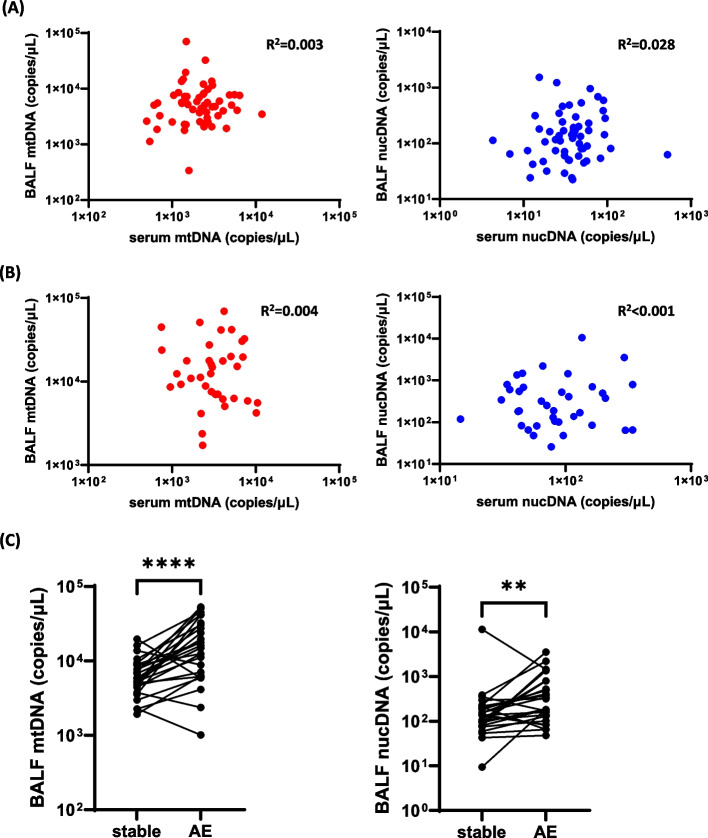
Correlation of target genes in BALF with those in serum and the occurrence of AE. **A**, **B** Copy numbers of target genes in BALF and serum were not correlated significantly each other either (**A**) in a stable state (*n* = 57) or (**B**) at the time of first AE (*n* = 36), determined by ddPCR. **C** mtDNA and nucDNA levels in BALF determined by ddPCR were significantly increased at the time of the first AE compared with those in a stable state (*n* = 27). Medians and interquartile ranges for the values of each target were: mtDNA, stable: 5575.7 (4478.6 – 8511.4) vs. AE: 15,441.4 (6467.1 – 29,678.6) copies/µL; nucDNA, stable: 137.1 (79.7 – 201.0) vs. AE: 318.9 (134.1 – 660.0) copies/µL. BALF: bronchoalveolar lavage fluid; mtDNA: mitochondrial DNA; nucDNA: nucleolar DNA, AE acute exacerbation; ddPCR: droplet-digital polymerase chain reaction; ** *P* < 0.01; **** *P* < 0.0001

Table 1 shows patient characteristics at the time of BAL. Among patients who provided both BALF and serum samples at the same time, we found no significant correlations between mtDNA and nucDNA levels, either in a stable state or at the time of AE (Fig. 1A, B). Among those who provided BALF samples both in a stable state and at the time of AE, BALF-mtDNA and BALF-nucDNA levels were significantly increased at the time of AE compared to those in a stable state (Fig. 1C).

**Table 1:** Patient characteristics at the time of BALF collection

Stable state, *N* = 90		At the time of the first AE, *N* = 38	
Age, years	67 (63–72)	Age, years	69 (64–75)
Sex, male	73 (80%)	Sex, male	36 (92%)
BMI, kg/m^2^	23.4 (21.1–25.6)	BMI, kg/m^2^	22.7 (21.5–24.9)
Smoking, ever	59 (65%)	Smoking, ever	38 (97%)
Smoking, pack-year	29.5 (0.0–46.8)	Smoking, pack-year	35.0 (2.3–52.9)
BALF volume, mL	76.8 (59.6–88.8)	BALF volume, mL	57.0 (43.5–72.5)
Total cell count, × 10^5^/μL	1.37 (0.87–2.36) ^*n*=89^	Total cell count, × 10^5^/μL	2.00 (1.32–2.86)
Neutrophils, %	0.8 (0.0–2.6) ^*n*=89^	Neutrophils, %	12.4 (3.4–26.9)
Lymphocytes, %	4.4 (1.6–9.6) ^*n*=89^	Lymphocytes, %	10.8 (3.5–16.6)
Eosinophils, %	0.4 (0.0–1.6) ^*n*=89^	Eosinophils, %	1.2 (0.4–2.8)
FVC, % predicted	79.7 (68.3–90.0)	FVC, % predicted	70.7 (57.1–79.9)
D_L_CO, % predicted	60.2 (46.3–72.5) ^*n*=88^	D_L_CO, % predicted	49.3 (36.8–61.5) ^*n*=37^
PaO_2_/FiO_2_	384.1 (329.5–419.6)	PaO_2_/FiO_2_	257.4 (217.9–315.0) ^*n*=36^
CRP, mg/dL	0.15 (0.06–0.63)	CRP, mg/dL	5.49 (3.10–11.15)
LDH, mg/dL	219 (196–250)	LDH, mg/dL	313 (283–373)
KL-6, U/mL	1220 (822–1958)	KL-6, U/mL	1484 (1134–1915) ^*n*=34^

Values are presented as median (interquartile range) or N (%). *BMI* Body mass index, *BALF* Bronchoalveolar lavage fluid, *AE* Acute exacerbation, *FVC* Forced vital capacity, *D*_*L*_*CO* Diffusing capacity of lung for carbon monoxide, *PaO*_*2*_ Partial pressure of arterial oxygen, *FiO*_*2*_ Fraction of inspiratory oxygen, *CRP* C-reactive protein, *LDH* Lactate dehydrogenase, *KL-6* Krebs von den Lungen-6

We explored the clinical variables correlated with the levels of BALF-mtDNA and -nucDNA. In a stable state, cigarette smoke exposure (pack-year), D_L_CO, and PaO_2_/FiO_2_ showed a negative correlation with BALF-mtDNA levels, while a positive correlation was observed with serum Krebs von den Lungen-6 (KL-6) levels (Table 2). BALF-nucDNA levels were negatively correlated with D_L_CO, FVC, and PaO_2_/FiO_2_ in a stable state, but positively correlated with BALF neutrophil and eosinophil rates, and serum levels of C-reactive protein (CRP), lactate dehydrogenase (LDH), and KL-6 (Table 3). On the other hand, at the time of the first AE, BALF-mtDNA levels were positively correlated with BALF neutrophil rates and negatively with age (Table 2). However, there were no significant correlations between BALF-nucDNA levels and clinical variables except for serum LDH levels (Table 3).

**Table 2:** Correlation of BALF-mtDNA and other clinical variables

**Stable state,** ***n*** **= 90**			**At the time of the first AE,** ***n*** **= 38**		
	**Spearman’s ρ**	***P***		**Spearman’s ρ**	***P***
Age, years	0.09	0.42	Age, years	-0.41	0.011*
BMI, kg/m^2^	0.07	0.51	BMI, kg/m^2^	-0.23	0.16
Smoking, pack-year	-0.33	0.002*	Smoking, pack-year	-0.13	0.43
BALF volume, mL	0.15	0.17	BALF volume, mL	-0.23	0.18
Total cell count, /μL	0.14	0.18	Total cell count, /μL	0.11	0.53
Neutrophils, %	0.06	0.55	Neutrophils, %	0.53	< 0.001*
Lymphocytes, %	-0.09	0.40	Lymphocytes, %	-0.32	0.052
Eosinophils, %	0.12	0.28	Eosinophils, %	0.07	0.70
FVC, % predicted	-0.20	0.06	FVC, % predicted	-0.32	0.051
D_L_CO, % predicted	-0.29	0.005*	D_L_CO, % predicted	-0.06	0.73
PaO_2_/FiO_2_	-0.34	0.0011*	PaO_2_/FiO_2_	0.10	0.55
CRP, mg/dL	0.13	0.23	CRP, mg/dL	0.17	0.30
LDH, mg/dL	0.10	0.34	LDH, mg/dL	-0.10	0.54
KL-6, U/mL	0.28	0.008*	KL-6, U/mL	0.08	0.66
	**mtDNA, copies/μL**	***P***		**mtDNA, copies/μL**	***P***
Sex, male	5027 (3195–7676)	0.20	Sex, male	12,429 (6643–25,586)	0.54
female	5897 (4543–9240)		female	14,846 (11,841–33,909)	
Smoking, ever	4740 (2529–7579)	0.006*	Smoking, ever	10,886 (6214–21,814)	0.23
never	6107 (4337–9444)		never	14,846 (11,773–41,614)	

For categorical variables, values are presented as median (interquartile range). *P*-values with * are statistically significant after false discovery rate adjustment for multiple comparison. *BMI* Body mass index, *BALF* Bronchoalveolar lavage fluid, *mtDNA* Mitochondrial DNA, *AE* Acute exacerbation, *FVC* Forced vital capacity, *D*_*L*_*CO* Diffusing capacity of lung for carbon monoxide, *PaO*_*2*_ Partial pressure of arterial oxygen, *FiO*_*2*_ Fraction of inspiratory oxygen, *CRP* C-reactive protein, *LDH* Lactate dehydrogenase, *KL-6* Krebs von den Lungen-6

**Table 3:** Correlation of BALF-nucDNA and other clinical variables

**Stable state,** ***n*** **= 91**			**At the time of the first AE,** ***n*** **= 38**		
	**Spearman’s ρ**	***P***		**Spearman’s ρ**	***P***
Age, years	0.20	0.06	Age, years	-0.14	0.41
BMI, kg/m^2^	0.09	0.39	BMI, kg/m^2^	-0.03	0.87
Smoking, pack-year	0.004	0.97	Smoking, pack-year	-0.01	0.95
BALF volume, mL	0.01	0.89	BALF volume, mL	-0.12	0.47
Total cell count, /μL	0.20	0.06	Total cell count, /μL	0.01	0.97
Neutrophils, %	0.42	< 0.001*	Neutrophils, %	0.09	0.59
Lymphocytes, %	-0.004	0.97	Lymphocytes, %	0.01	0.97
Eosinophils, %	0.45	< 0.001*	Eosinophils, %	-0.09	0.61
FVC, % predicted	-0.36	< 0.001*	FVC, % predicted	0.03	0.87
D_L_CO, % predicted	-0.30	0.005*	D_L_CO, % predicted	-0.04	0.83
PaO_2_/FiO_2_	-0.43	< 0.001*	PaO_2_/FiO_2_	-0.21	0.22
CRP, mg/dL	0.24	0.02*	CRP, mg/dL	0.20	0.22
LDH, mg/dL	0.35	< 0.001*	LDH, mg/dL	0.33	0.045*
KL-6, U/mL	0.42	< 0.001*	KL-6, U/mL	-0.03	0.85
	**nucDNA, copies/μL**	***P***		**nucDNA, copies/μL**	***P***
Sex, male	106.3 (60.4–191.8)	0.70	Sex, male	187.7 (93.0–694.3)	0.52
female	155.6 (64.3–219.0)		female	488.6 (415.3–505.7)	
Smoking, ever	106.3 (63.0–205.7)	0.59	Smoking, ever	186.9 (93.0–644.1)	0.34
never	146.6 (76.3–242.4)		never	408.0 (178.7–398.6)	

For categorical variables, values are presented as median (interquartile range). *P*-values with * are statistically significant after false discovery rate adjustment for multiple comparison. *BMI* Body mass index, *BALF* Bronchoalveolar lavage fluid, *nucDNA* Nucleolar DNA, *AE* Acute exacerbation, *FVC* Forced vital capacity, *D*_*L*_*CO* Diffusing capacity of lung for carbon monoxide, *PaO*_*2*_ Partial pressure of arterial oxygen, *FiO*_*2*_ Fraction of inspiratory oxygen, *CRP* C-reactive protein, *LDH* Lactate dehydrogenase, *KL-6* Krebs von den Lungen-6

Thus, in a stable state, both BALF-mtDNA and BALF-nucDNA levels were found to be correlated with the degree of lung function impairment and inflammation, whereas cigarette smoking was correlated only with BALF-mtDNA levels. At the time of AE, it showed significant correlation with BALF neutrophils.

### BALF-mtDNA and survival of IPF

**Fig. 2:**
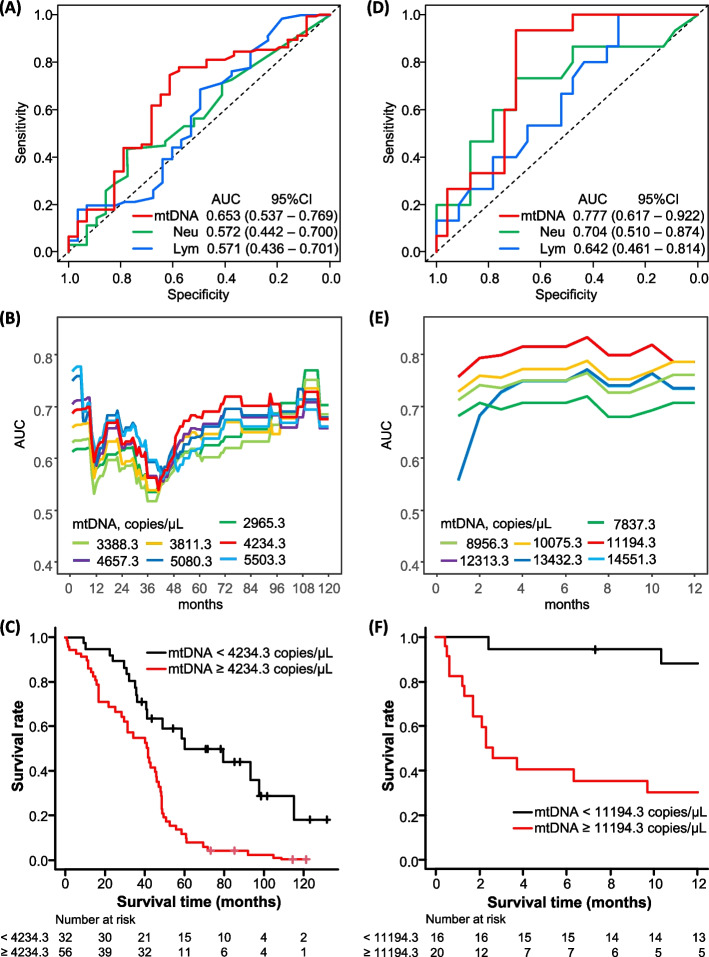
Survival prediction by BALF-mtDNA. **A** ROC curves for BALF-mtDNA, neutrophil rates, and lymphocyte rates for predicting 5-year mortality from a stable state. **B** Changes in AUC values over time from a stable state for each cutoff value of BALF-mtDNA. **C** Patients with a higher BALF-mtDNA showed shorter survival time than those with a low BALF-mtDNA in a stable state after adjusting for age, sex, body mass index, and baseline forced vital capacity and diffusing capacity of lung for carbon monoxide (median survival time: 42.4 months in BALF-mtDNA ≥ 4234.3 copies/µL vs. 79.6 months in BALF-mtDNA < 4234.3 copies/µL, *p* < 0.001). **D** ROC curves for BALF-mtDNA, neutrophil rates, and lymphocyte rates for predicting 6-month mortality from the time of AE. Since higher lymphocyte rates in BALF at the time of AE tended to be associated with longer survival, the lymphocyte rate values were transformed into negative numbers before conducting the time-dependent ROC analysis. **E** Changes in AUC values over time from the time of AE for each cutoff value of BALF-mtDNA. **F** Patients with a higher BALF-mtDNA showed poorer survival times than those with a low BALF-mtDNA at the time of first AE after adjusting for age, sex and partial pressure of arterial oxygen/fraction of inspiratory oxygen (median survival time: 2.6 months in BALF-mtDNA ≥ 11,194.3 copies/µL vs. 20.0 months in BALF-mtDNA < 11,194.3 copies/µL, *p* = 0.03). Baseline FVC and D_L_CO data were recorded within 6 months prior to the AE. AUC: area under the curve; 95%CI: 95% confidence interval; mtDNA: mitochondrial DNA; Neu: neutrophils; Lym: lymphocytes; ROC: receiver operating characteristic

We investigated the prognostic significance of BALF-mtDNA in comparison with the rates of neutrophils and lymphocytes in BALF, which have been reported as prognostic factors [14, 15]. Using time-dependent ROC analyses, we revealed a relatively better prognostic value of BALF-mtDNA than that of the rates of neutrophils or lymphocytes in BALF (Fig. 2A). A BALF-mtDNA of 4234.3 copies/µL and a BALF-nucDNA of 78.0 copies/µL in a stable state were identified to predict 5-year mortality (Fig. 2B and Figure S2A). After adjusting for age, sex, body mass index (BMI), and baseline FVC and D_L_CO, BALF-mtDNA ≥ 4234.3 copies/µL in a stable state was significantly correlated with survival time (Fig. 2C and Table 4, MST: 79.6 vs. 43.1 months), while BALF-nucDNA ≥ 78.0 copies/µL was not.

**Table 4:** Prognostic values of BALF-mtDNA and nucDNA

	Sensitivity	Specificity	Hazard ratio (95%CI)	*P*
BALF in a stable state				
mtDNA ≥ 4234.3 copies/µL	0.747	0.613	3.136 (1.735–5.669)	< 0.001
nucDNA ≥ 78.0 copies/µL	0.933	0.696	1.430 (0.779–2.625)	0.25
BALF at the time of the first AE				
Model 1				
mtDNA ≥ 11,194.3 copies/µL	0.737	0.486	2.837 (1.191–6.759)	0.02
nucDNA ≥ 602.6 copies/µL	0.400	0.826	1.047 (0.410–2.676)	0.92
Model 2				
mtDNA ≥ 11,194.3 copies/µL			2.479 (1.113–5.524)	0.03
nucDNA ≥ 602.6 copies/µL			1.063 (0.441–2.567)	0.89
Model 3				
mtDNA ≥ 11,194.3 copies/µL			2.533 (1.123–5.715)	0.03
nucDNA ≥ 602.6 copies/µL			1.014 (0.414–2.485)	0.98
Model 4				
mtDNA ≥ 11,194.3 copies/µL			2.513 (1.081–5.843)	0.03
nucDNA ≥ 602.6 copies/µL			1.411 (0.557–3.572)	0.47

Sensitivities and specificities in a stable state are for predicting 5-year mortality, while those at the time of the first AE are for predicting 6-month mortality. Hazard ratios for a stable state were adjusted for age, sex, body mass index, and baseline FVC and D_L_CO. Hazard ratios for the time of AE were adjusted for age and sex, and partial pressure of arterial oxygen/fraction of inspiratory oxygen (Model 1), body mass index (Model 2), C-reactive protein (Model 3), or baseline FVC and D_L_CO (Model 4). *BALF* Bronchoalveolar lavage fluid, *mtDNA* Mitochondrial DNA, *nucDNA* Nucleolar DNA, *95%CI* 95% confidence interval, *BAL* Bronchoalveolar lavage, *AE* acute exacerbation, *FVC* Forced vital capacity, *D*_*L*_*CO* Diffusing capacity of lung for carbon monoxide

### BALF-mtDNA and survival from AE-IPF

Because there is no firm predictor for survival of AE-IPF to date, we conducted further analyses to evaluate correlation of BALF-mtDNA at the time of AE-IPF diagnosis with the prognosis. Time-dependent ROC analyses revealed a relatively better prognostic value of BALF-mtDNA than that of the rates of neutrophils or lymphocytes in BALF (Fig. 2D). A BALF-mtDNA level of 11,194.3 copies/µL and a BALF-nucDNA level of 602.6 copies/µL at the time of AE were identified as optimal cut-off values to predict 6-month mortality (Fig. 2E and Figure S2B). BALF-mtDNA ≥ 11,194.3 copies/µL at the time of AE was significantly correlated with survival time after the diagnosis of AE, even after adjusting for age, sex, and PaO_2_/FiO_2_ (Fig. 2F and Table 4, MST: 30.3 vs. 2.3 months). The results were similar when using BMI, serum CRP levels, or FVC and D_L_CO values recorded within 6 months before the diagnosis of AE, as adjusting variables instead of PaO_2_/FiO_2_ (Table 4). It should be noted that this cut-off value sharply distinguished survivors from non-survivors. Other variables such as BALF-nucDNA, BALF neutrophil ratios, PaO_2_/FiO_2_, and serum biomarkers shown in Table 2, did not show a significant correlation with survival time. Serum-mtDNA in BALF collected at the time of AE, which did not correlate with BALF-mtDNA, also did not show a correlation with survival time (see the Results in the Supporting Information).

## Discussion

Our study revealed relationships between BALF-mtDNA and lung function, smoking status, inflammatory cells in BALF, and prognosis in patients with IPF. Intriguingly, the results suggested that increased BALF-mtDNA can be a potential indicator of poor prognosis for IPF, being associated with survival time in both stable and acute phases. In particular, it clearly distinguished survivors from non-survivors in patients with AE-IPF in our cohort. Meanwhile, BALF-nucDNA did not show prognostic value in either phase. This is the first study to demonstrate the clinical significance of BALF-mtDNA on IPF, which was measured precisely using ddPCR.

mtDNA is known as a DAMP that triggers inflammatory responses via pathogen recognition receptors, such as Toll-like receptor-9 [16]. It can be released outside mitochondria through various mechanisms, including cell death and other mechanisms unrelated to cell death (active release), such as mtDNA damage and oxidative stress caused by reactive oxygen species [17–19]. In the field of IPF, several studies have evaluated the prognostic impact of plasma/serum-mtDNA. Extracellular mtDNA was elevated in plasma or serum from IPF patients, and was associated with shorter survival [3, 7], disease progression [3, 5, 7], future development of AE-IPF [6], and clinical parameters representing disease severity, such as lower D_L_CO and shorter 6-min walk distance [4, 7]. On the other hand, Bruno et al. demonstrated elevated BALF-mtDNA in IPF [4], although its clinical significance and prognostic impact have not been studied in detail, and how BALF-mtDNA levels alter depending on the disease status has never been investigated. In our study, levels of BALF-mtDNA and serum-mtDNA were poorly correlated with each other in both stable and acute phases. While the levels of BALF-mtDNA are expected to reflect the concentration of mtDNA in the epithelial lining fluid of the lungs, its kinetics, such as half-life, have seldom been studied. On the other hand, the kinetics of mtDNA levels in the bloodstream have been studied in various acute disease states, and its half-life is expected to be short. Circulating mtDNA levels are suggested to be influenced by multiple systemic parameters, including the concentration of DNase in the blood and clearance by organs such as the liver and kidney. Based on these results, we speculate that BALF-mtDNA may sensitively reflect local lung damage and be elevated even in mild disease, while serum-mtDNA may be elevated in more advanced disease and reflect systemic inflammation. To elucidate these hypotheses, further studies are warranted.

Clinical parameters such as baseline lung function [14, 20–23], worse oxygenation [14, 24], neutrophils and lymphocytes in BALF [14, 15], and blood biomarkers like CRP [14], LDH [24, 25], and KL-6 [24] have been identified as prognostic factors of AE-IPF, though their impact has varied between studies. Our study results may provide new insights into this area. Given that the MST of AE-IPF is generally recognized as 3–4 months [2, 26], our findings indicate that BALF-mtDNA can distinguish between AE-IPF patients with an MST of less than 3 months (non-survivors) and those with an MST of more than 30 months (survivors). Meanwhile, serum-mtDNA was not correlated with survival after the diagnosis of AE-IPF, though the number of the assessed serum samples was limited. This suggests that the dynamics of mtDNA circulating in the local lung are distinct from that in the systemic condition in the acute setting. Given that BALF-mtDNA, but not nucDNA, which is generally released during cell death, was significantly correlated with BALF-neutrophils, active release of mtDNA from cells may be related to acute neutrophilic inflammation.

In stable IPF, age, sex, baseline FVC and D_L_CO are widely accepted prognostic factors [27]. Meanwhile, though there are a few reports insisting on the prognostic value of BALF biomarkers such as surfactant protein A and neutrophils [28, 29], supporting evidence for them is still lacking. Our study found that BALF-mtDNA was associated with survival of IPF in a stable state. Additionally, it is worth noting that the disease severity in our cohort was relatively mild, as evidenced by a median FVC of approximately 80% predicted. Therefore, BALF-mtDNA may be a sensitive factor correlating with survival, even in the early stages of the disease with preserved lung function.

In this study, BALF-mtDNA levels in a stable state were correlated with the severity of lung function impairment (reduced D_L_CO and PaO_2_/FiO_2_) as well as serum KL-6, which has been reported to reflect the extent of lung involvement in IPF. Similar correlations were also observed with BALF-nucDNA. The increases in both BALF-mtDNA and BALF-nucDNA may reflect the increased apoptosis in the lungs. Meanwhile, it is worth noting that, unlike BALF-nucDNA, BALF-mtDNA levels were significantly decreased in smokers compared to non-smokers. A previous study has revealed increased mtDNA damage and deletion, as well as decreased mtDNA in macrophages in BALF from smokers compared with that from non-smokers [30]. While a number of other studies have demonstrated increased mtDNA levels in blood [18] or cells exposed to cigarette smoke [31, 32], the alteration of extracellular mtDNA levels in BALF has not been extensively studied. Although further studies investigating the effect of smoking on BALF-mtDNA are warranted, our data suggest that the increased BALF-mtDNA observed in IPF patients may reflect the pathological mechanisms correlated with disease severity and progression, rather than non-specific oxidative stress increased in the lungs of smokers.

ddPCR is a highly sensitive gene detection method increasingly used in medical and basic research. Moreover, this novel method has the great advantage of simplifying the sample preparation steps by using crude samples. It has been applied to measure various biomarkers in BALF and bronchial washing fluid, such as lung microbiota [33–36] and pathogenic gene mutations in lung cancer [37]. However, ddPCR has not been used to detect biomarkers of pulmonary fibrosis, and no previous study has evaluated mtDNA levels in BALF using ddPCR. We believe that the preciseness and result reproducibility of ddPCR strengthen the robustness of our findings, and the convenience supports its future application to both clinical practice and basic research.

Some limitations of this study should be acknowledged. First, it is a retrospective study with a limited number of included cases conducted in a single centre without a validation cohort. Therefore, further research is needed to confirm the results. Second, our stable IPF cohort included mainly mild cases, so it is unclear whether our findings can be applied to more severe cases. However, our study suggests that BALF-mtDNA may be prognostic at least in mild cases. Third, due to the invasiveness of BAL and limited diagnostic information which can be obtained from it, it is not routinely recommended for evaluating IPF/AE-IPF. While measuring mtDNA may extend the usefulness of BAL, a less invasive procedure for obtaining samples reflecting information in the alveolar lining fluid is expected and would further enhance the clinical value of mtDNA as a prognostic biomarker. Fourth, due to the lack of information regarding comorbidities, this report could not provide any information regarding the correlation between patients’ comorbidities and BALF-mtDNA levels. Fifth, due to the ethical limitation related with the invasiveness of BAL procedure, we could not obtain BALF samples from healthy controls. This made interpretation of our findings challenging. Lastly, the limited number of patients who provided serum samples may have led to an underestimation of the prognostic impact of serum mtDNA at the time of AE. Further study is needed to reach a conclusion.

## Conclusions

Elevated BALF-mtDNA levels were associated with inflammation or disordered pulmonary function in a stable state, and with an elevated neutrophil ratio in BALF at the time of AE. They were also associated with shorter survival in both stable IPF and at the time of AE. Notably, higher BALF-mtDNA levels may help distinguish non-survivors from survivors in AE-IPF. Given the trends shown by analyses for serum-mtDNA and BALF-nucDNA, the elevation of BALF-mtDNA might reflect different underlying aetiology reflected by serum-mtDNA and might not simply reflect the impact of cell collapse. Further research is required to explore the underlying mechanisms and clinical applications of BALF-mtDNA in IPF.

### Supplementary Information

Additional file 1. Additional methods regarding processing of BALF and blood, and ddPCR, additional results regarding optimisation of ddPCR protocol and correlation between serum-mtDNA and survival from AE-IPF, and Figure S1 and S2.

## Data Availability

The datasets used and/or analysed during the current study are available from the corresponding author on reasonable request.

## Abbreviations

AE: Acute exacerbation
AUC: Area under the curve
BAL: Bronchoalveolar lavage
BALF: Bronchoalveolar lavage fluid
BMI: Body mass index
CRP: C-reactive protein
DAMPs: Danger-associated molecular pattern molecules
ddPCR: Droplet digital polymerase chain reaction
D_L_CO: Diffusing capacity of the lung for carbon monoxide
FiO_2_: Fraction of inspiratory oxygen
FVC: Forced vital capacity
IPF: Idiopathic pulmonary fibrosis
LDH: Lactate dehydrogenase
MST: Median survival time
mtDNA: Mitochondrial deoxyribonucleic acid
nucDNA: Nucleolar deoxyribonucleic acid
PaO_2_: Partial pressure of arterial oxygen
PCR: Polymerase chain reaction
ROC: Receiver operating characteristic
