# Supplementary material for: Mitochondrial DNA in bronchoalveolar lavage fluid is associated with the prognosis of idiopathic pulmonary fibrosis: a single cohort study
Source: Respir Res. 2024 May 10;25:202. doi: 10.1186/s12931-024-02828-9 (PMC11083749; doi:10.1186/s12931-024-02828-9)
Supplement: Supplementary file 1 — Additional file 1. Additional methods regarding processing of BALF and blood, and ddPCR, additional results regarding optimisation of ddPCR protocol and correlation between serum-mtDNA and survival from AE-IPF, and Figure S1 and S2. [file 12931_2024_2828_MOESM1_ESM.docx]

**Supporting information**

**Mitochondrial DNA in bronchoalveolar lavage fluid is associated with the prognosis of idiopathic pulmonary fibrosis: a single cohort study**

Jun Fukihara, Koji Sakamoto, Yoshiki Ikeyama, Taiki Furukawa, Ryo Teramachi, Kensuke Kataoka, Yasuhiro Kondoh, Naozumi Hashimoto, and Makoto Ishii

# Methods

*Processing of bronchoalveolar lavage fluid (BALF) and blood*

The fluid was filtered using two layers of surgical gauze and centrifuged at 300 g for 5 min at 4℃. The resulting cell pellet was used for differential cell counts by staining ≥300 cells with Diff-Quick^TM^ stain (Scientific Products, McGraw Park, IL). Blood samples were also centrifuged at 200 g for 5 minutes to obtain serum. The serum obtained and the supernatant from BALF were stored at -80℃ for extracellular DNA extraction.

*Droplet-digital polymerase chain reaction (ddPCR)*

For crude extracellular DNA extraction and sample preparation, BALF supernatant and serum samples were mixed with QuickExtract^TM^ DNA Extraction Solution (Biosearch Technologies, Hoddesdon, UK) at a ratio of 1:2 and heated at 65℃ for 6 minutes and subsequently at 98℃ for 2 minutes. An alternative column-based DNA purification method was also used, using a QIAamp MiniElute Virus Spin Kit (Quiagen, Hilden, Germany) and Plasma/Serum Cell-Free Circulating DNA Purification Mini Kit (Norgen Biotek, Thorold, ON, Canada) for BALF and serum samples according to the manufacturer’s protocols, respectively. The DNA concentrations of the two methods were compared to ensure validity and consistency.

For ddPCR, a reaction solution of 22 µL was made up with 11 µL of ddPCR Supermix for Probes (Bio-Rad Laboratories, Hercules, CA, USA), 1.1 µL each of a custom-made FAM-tagged human mitochondrial NADH-ubiquinone oxidoreductase chain 1 (mt-ND1) probe (forward: ACATTACTAATAAGTGGCTCC, reverse: GAGTAATCAGAGGTGTTCTT, Bio-Rad Laboratories), a HEX-tagged human ribonuclease P protein subunit p30 (RPP30) probe (UniqueAssayID: dHsaCP2500350, Bio-Rad Laboratories), and 8.8 µL of diluted DNA extract solution prepared with nuclease-free water. The copy numbers of mt-ND1 and RPP30 served as markers for extracellular mtDNA and nucleolar DNA (nucDNA), respectively.

We used the QX200 AutoDG Droplet Digital PCR system (Bio-Rad Laboratories, Hercules, CA, USA) for ddPCR according to the manufacturer’s protocol. From the 22 µL reaction solution, 20 µL was taken and divided into 20,000 droplets. PCR reactions were conducted with hot-start activation at 94℃ for 10 minutes, followed by 40 cycles of PCR at 94℃ for 30 seconds and 53℃ for 60 seconds. The number of droplets containing at least 1 copy of the target DNA fragment was counted using the droplet reader, and the concentration (copies/µL reaction solution) of the target was calculated using QuantaSoft^TM^ software (Bio-Rad Laboratories) by fitting the fraction of positive droplets to a Poisson algorithm. The concentration in the original BALF and serum samples was calculated by taking into account the sample dilution during reaction solution preparation and DNA extraction.

# Results

*Optimisation of ddPCR protocol*

Two DNA extraction protocols were compared for ddPCR analysis of both BALF and serum samples. The QuickExtract^TM^ protocol using crude samples detected higher concentrations of target DNA fragments more quickly than the standard kit-based protocol while maintaining the magnitude of correlations (Figure S1A, B). Given these results and recent reports demonstrating the usefulness of ddPCR using crude samples (9), we used the QuickExtract^TM^ protocol for both BALF and serum samples in further experiments.

Although ddPCR is more sensitive and accurate than conventional quantitative PCR, some samples exhibit very low BALF-nucDNA copy numbers depending on the extent of sample dilution. Results were not consistent when the concentration was <1.5 copies/μL (Figure S1C). A dose-setting study showed that a 1:7.5–8.5 dilution of the original BALF was optimal for achieving a sufficiently high concentration of nucDNA, while keeping the mtDNA concentration, which was approximately 10 times higher than that of nucDNA, below the extremely high levels at which the dose-concentration curve plateaus (Figure S1D). This dilution resulted in a maximum concentration achievable through our protocol of 7–8 μL DNA extract solution/20 μL reaction solution, which enabled us to measure both mtDNA and nucDNA in all samples.

*Serum-mtDNA and survival from AE-IPF*

Using time-dependent receiver operating characteristic analyses, a serum mitochondrial DNA (mtDNA) level of 2520.0 copies/µL and a nucleolar DNA (nucDNA) level of 36.0 copies/µL to predict 6-month survival were identified as optimal cut-off values (sensitivity, specificity and area under the curve (AUC) of serum-mtDNA and -nucDNA were 0.714, 0.409 and 0.471, and 1.000, 0.182 and 0.541, respectively). However, even at those optimal cut-off values, serum-mtDNA and nucDNA at the time of the first acute exacerbation (AE) were not correlated with survival time after the diagnosis of AE, both before (data not shown) and after adjusting for age, sex, and partial pressure of arterial oxygen/fraction of inspiratory oxygen that were recorded within 6 months before the diagnosis of AE (for a cut-off of mtDNA ≥2794.3 copies/µL, hazard ratio: 1.030; 95% confidence interval: 0.460–2.307; p=0.94. For a cut-off of nucDNA ≥42.0 copies/µL, hazard ratio: 1.826; 95% confidence interval: 0.468–7.123; p=0.39).

**
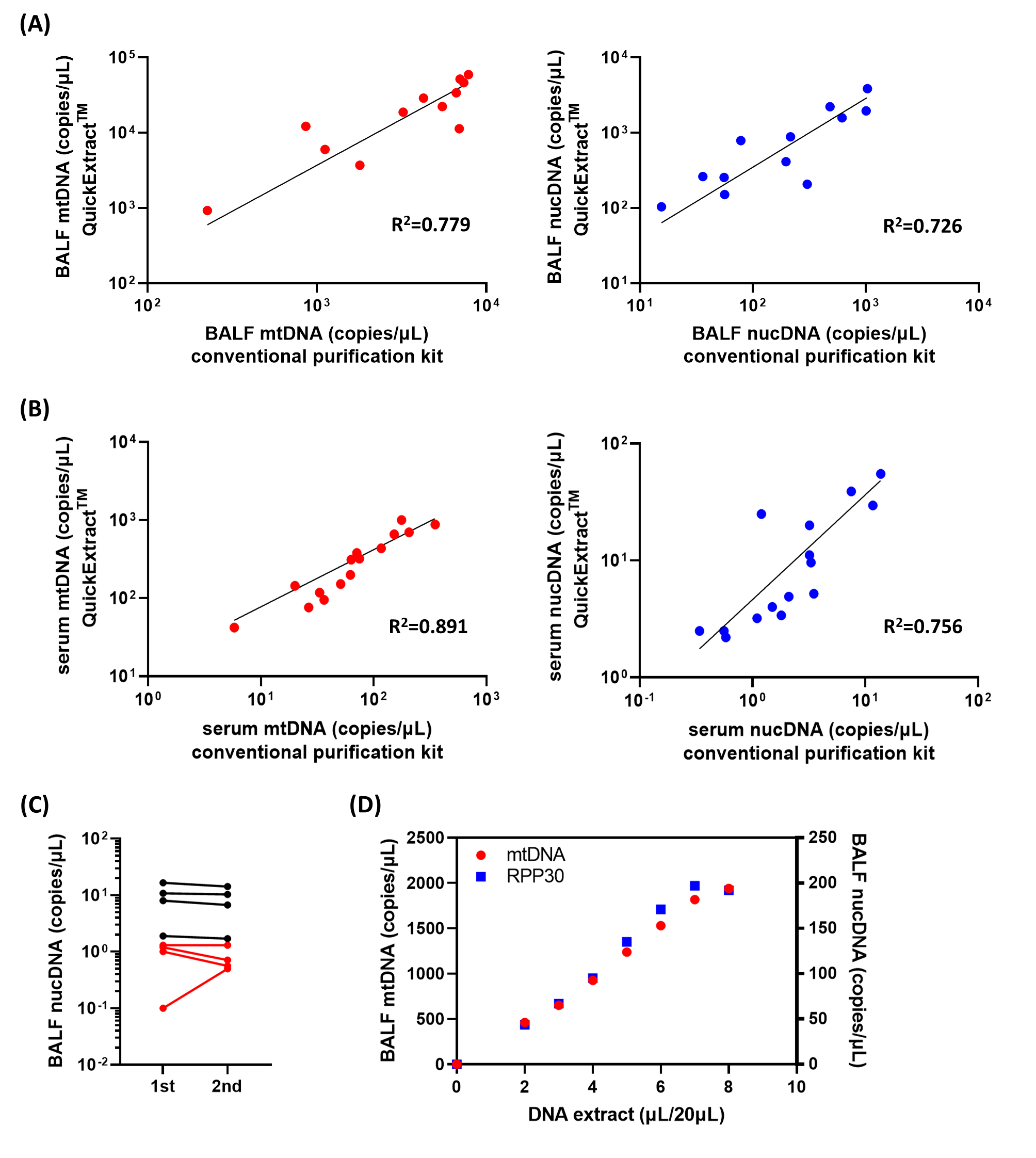
Figure S1**

Optimization of ddPCR for BALF and serum. (A) The copy numbers of target DNA fragments measured by ddPCR using DNA templates purified from BALF using QuickExtract^TM^ DNA Extraction Solution and a conventional kit (QIAamp MiniElute Virus Spin Kit) were well correlated linearly, and their magnitude correlation was preserved (n=13). (B) The copy numbers of target genes measured by ddPCR using DNA templates purified from serum using QuickExtract^TM^ DNA Extraction Solution and a conventional purification kit (Plasma/Serum Cell-Free Circulating DNA Purification Mini Kit) were well correlated linearly and their magnitude of correlation was preserved (n=15). (C) BALF-nucDNA copy numbers were quantified twice using the same samples (n=8) by ddPCR in the same experimental condition. The results from the samples with nucDNA values less than 1.5 copies/μL are presented as red dots and lines, showing that data from those samples vary more than that from the samples with higher nucDNA values. (D) The copy numbers of BALF-mtDNA and nucDNA measured by ddPCR increased linearly as the volumes of DNA extract solution in the PCR reaction solution increased. ddPCR: droplet-digital polymerase chain reaction; BALF bronchoalveolar lavage fluid; mtDNA: mitochondrial DNA; nucDNA: nucleolar DNA.

Figure S2


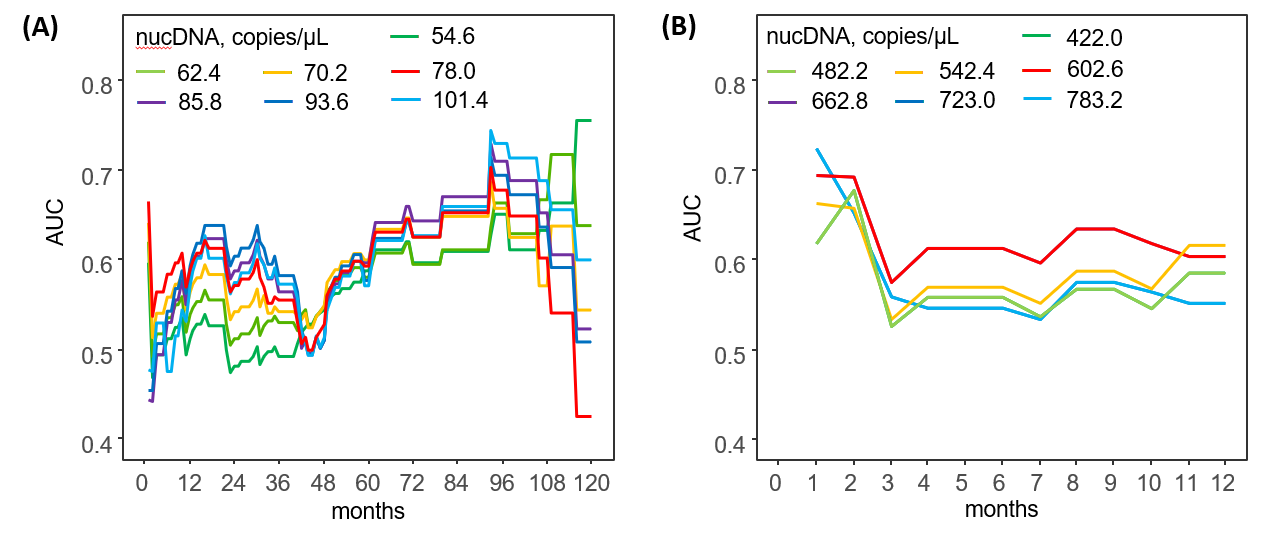


Changes in AUC values over time from a stable state (A) or the time of AE (B) for each cutoff value of BALF-nucDNA. AUC: area under the curve; AE: acute exacerbation; BALF: bronchoalveolar lavage fluid; nucDNA: nucleolar DNA.
